# Retraction: High-Affinity Naloxone Binding to Filamin A Prevents Mu Opioid Receptor–Gs Coupling Underlying Opioid Tolerance and Dependence

**DOI:** 10.1371/journal.pone.0266627

**Published:** 2022-03-30

**Authors:** The PLOS ONE Editors

Following the publication of this article [1], concerns were raised regarding results presented in Figs 1 and 7. Specifically,

- There appear to be horizontal and vertical irregularities suggestive of splice lines in the following panels:
  ○ Between lanes between lanes 4–5 of the Fig 1A left and right FLNA panels, right MOR panel, and left and right Gα panels.
  ○ Between the 92.3kDa and the 50.4kDa marker of the Fig 1A left MOR panel.
  ○ Between lanes 2–3 of the Fig 7A Morphine + NLX +FLNA_2550-2560_ panel.
  ○ Around multiple bands presented in the Fig 7A MOR and Gα panels
- In Fig 1C, neither the published panels nor the underlying data provided in follow-up discussions include a positive control sample. The absence of a positive control calls into question the reliability of the results presented in Fig 1C.
- The Fig 7A NLX and FLNA_2550-2560_ panels appear similar.

The corresponding author noted that the Fig 7A NLX and FLNA_2550-2560_ panels were inadvertently duplicated and provided a replacement panel for the FLNA_2550-2560_ panel. However, the corresponding author disagreed with the Fig 1A concerns, stating that the observations are likely the result of image compression artifacts.

The corresponding author provided image data to support the contested western blot results in this [1] and other PLOS ONE articles [2–5]. Per PLOS’ assessment of the data files, the pixel patterns in background areas of blot images provided for multiple panels in [1–5] appear more similar than would be expected for data obtained in independent experiments. Furthermore, the supporting data files did not contain positive controls as needed to verify the reliability of the results. In response to these concerns, the corresponding author stated that the repetitive features in the background noise of the image data are likely the result of scanner artifacts. The explanation given for the background image similarities does not resolve the journal’s concerns in light of PLOS’ assessment of the data files.

The data and comments provided did not resolve the concerns about the integrity and reliability of data presented in this article. In light of these issues, the *PLOS ONE* Editors retract this article.

HYW and LB did not agree with the retraction. MF either did not respond directly or could not be reached. HYW stands by the article’s findings.
